# Genome-Wide Association Study Reveals Novel Loci for SC7 Resistance in a Soybean Mutant Panel

**DOI:** 10.3389/fpls.2017.01771

**Published:** 2017-10-11

**Authors:** Zhijun Che, Hailun Liu, Fanglei Yi, Hao Cheng, Yuming Yang, Li Wang, Jingyi Du, Peipei Zhang, Jiao Wang, Deyue Yu

**Affiliations:** ^1^National Key Laboratory of Crop Genetics and Germplasm Enhancement, National Center for Soybean Improvement, Nanjing Agricultural University, Nanjing, China; ^2^Key Laboratory of Plant Resources Conservation and Sustainable Utilization, South China Botanical Garden, Chinese Academy of Sciences, Guangzhou, China

**Keywords:** association analysis, soybean mosaic virus, linkage disequilibrium, soybean, mutant population

## Abstract

Soybean mosaic virus (SMV) is a member of *Potyvirus* genus that causes severe yield loss and destroys seed quality in soybean [*Glycine max* (L.) Merr.]. It is important to explore new resistance sources and discover new resistance loci to SMV, which will provide insights to improve breeding strategies for SMV resistance. Here, a genome-wide association study was conducted to accelerate molecular breeding for the improvement of resistance to SMV in soybean. A population of 165 soybean mutants derived from two soybean parents was used in this study. There were 104 SNPs identified significantly associated with resistance to SC7, some of which were located within previous reported quantitative trait loci. Three putative genes on chromosome 1, 9, and 12 were homologous to *WRKY72, eEF1Bβ*, and *RLP9*, which were involved in defense response to insect and disease in *Arabidopsis*. Moreover, the expression levels of these three genes changed in resistance and susceptible soybean accessions after SMV infection. These three putative genes may involve in the resistance to SC7 and be worthy to further research. Collectively, markers significantly associated with resistance to SC7 will be helpful in molecular marker-assisted selection for breeding resistant soybean accessions to SMV, and the candidate genes identified would advance the functional study of resistance to SMV in soybean.

## Introduction

Soybean [*Glycine max* (L.) Merr.] provides abundant protein for humans and is also one of the world important oil crops. Soybean mosaic virus (SMV) is a member of *Potyvirus* genus and causes severe yield loss and destroy seed quality (Adams et al., 2005). In the United States, according to disease reactions by resistant cultivars (Buffalo, Davis, Kwanggyo, Marshall, Ogden, and York) and two susceptible cultivars (Clark and Rampage), 98 isolates SMV strains had been classified into seven strains and named G1 to G7 (Cho and Goodman, 1979, 1982). Resistance loci, *Rsv1, Rsv3*, and *Rsv4* had been mapped to chromosomes 13, 14, and 2, respectively (Hayes et al., 2000, 2004; Gore et al., 2002; Wang et al., 2010). *Rsv1* in cultivar PI 96983 was resistant to G1–G6 but susceptible to G7 (Cho and Goodman, 1982); *Rsv3* was resistant to strains G5–G7 and *Rsv4* to G1–G7 (Hayes et al., 2000; Jeong et al., 2002). Based on SMV isolates reaction to a serious of soybean differentials, SMV had been classified into 21 strains in China and named SC1 to SC21 (Wang et al., 2003; Guo et al., 2005; Li et al., 2010). SC7 is a virulent strain that prevalent in Huang-Huai-Hai valleys in China (Wang et al., 2003). Yang et al. (2013) fine-mapped two dominant genes resistant to SC3, SC6, SC7, and SC17 near *Rsv1* locus. Wang et al. (2011) fine-mapped resistance gene to SMV strain SC8 in Kefeng No.1 which was in the neighbor of *Rsv4*. Yan et al. (2015) fine-mapped the resistance gene Rsc7 in Kefeng No.1 to a region of approximately 158 kb on chromosome 2 that is between SSR marker BARCSOYSSR_02_0621 and BARCSOYSSR_02_0632. Due to different hosts and cultivars use in identifying SMV strains, the relationship between SC1-SC21 in China and G1–G7 in United States is unknown.

Nucleotide binding sites (NBS) and leucine rich repeat (LRR) domain were contained in many plant disease resistance genes. There were 19 genes contain NBS-LRR domain in *Rsv1* locus and at least 10 different *Rsv1* alleles have been found in various landraces, such as *Rsv1* in PI 96983, *Rsv1*-d in FT-10, *Rsv1*-h in Suweon 97, *Rsv1*-k in Kwanggyo, *Rsv1*-m in Marshall, *Rsv1*-n in PI 507389, *Rsv1*-r in Raiden, *Rsv1*-s in LR1, *Rsv1*-t in Ogden, and *Rsv1*-y in York (Buss et al., 1989; Chen et al., 1991, 2001, 2002; Ma et al., 1995, 2003; Silva et al., 2004; Tucker et al., 2009; Shao et al., 2014). Many resistance gene loci were mapped by use of cross between narrow genetic resources based on resistance and susceptible accessions. Though this position method was effective, only limited gene loci have been mapped. Therefore, new method is needed to explore new resistant genes in soybean. Compared with linkage analysis, much more alleles and broader heritable diversities could be identified in genome-wide association analyses (GWAS) (Mackay, 2001).

Genome-wide association study is a powerful technique that enables researchers to exploit natural variations and locate candidate genes in the genome (Korte and Farlow, 2013). In recent years, with the development of the technology of sequencing, a large number of SNP markers were developed. Combining high-quality of SNP markers and phenotypic resources, GWAS has been successfully applied in many crops. In rice, a substantial number of loci were identified performed by GWAS for 14 agronomic traits that potentially important for rice production and improvement (Huang et al., 2010). In maize, Wang X. et al. (2016) used a GWAS of maize drought tolerance at the seedling stage and identified 83 genetic variants, which were resolved to 42 candidate genes. According to the SNP signal, *ZmVPP1* was identified contributing to this trait (Wang X. et al., 2016). In soybean, Zhang et al. (2014) combined genome-wide association analysis and linkage analysis identified *GmACP1* gene that related to P efficiency. Nineteen SNPs and five haplotypes for soybean yield components based on 1536 SNPs were identified by GWAS (Hao et al., 2012). Moreover, GWAS can provide a comparatively higher resolution in terms of mapping the genomic position of interested genes or QTLs because it can be applied to germplasm collections or naturally occurring populations (Shirasawa et al., 2013).

Resistance to SMV can be qualitative or quantitative. Qualitative resistance is controlled by a pair of single dominant genes and confers complete resistance to some SMV strains but susceptibility to others. Quantitative resistance is controlled by multiple genes in soybean (Zhi et al., 2005), is more broad-spectrum and durable than qualitative resistance, but it confers just partial resistance to SMV.

In our study, a genome-wide association analysis was performed to a diverse panel of 165 soybean mutants genotyped with the 355 K SoySNP array and phenotyped with resistance to SMV strain of SC7. The objective of this study was to identify candidate genes significantly associated with resistance in development to SC7. These results will be helpful for cloning of SC7 resistance genes and applying in molecule marker-assisting breeding.

## Materials and Methods

### Plant Materials and SMV Strains

Soybean mutant population was derived from two parental accessions Nannong 86-4 and Nannong 94-16 through compound chemical (ethyl methanesulfonate, EMS) and physical(^60^Coγ) mutagenesis (Han et al., 2007, 2008). After years of investigating of phenotypic traits, we finally obtained 165 soybean mutants, among which 93 mutants were from Nannong 86-4 and 72 mutants were from Nannong 94-16. These mutants have extensive variations in leaf, stem, flower, seed shape, protein, oil contents, etc. (Han et al., 2007, 2008). SMV strain SC7 was conserved on highly SMV susceptible cultivar Nannong 1138-2 and the viruliferous leaves were frozen in -80°C refrigerator. All the plant materials and SC7 strain were provided by the National Center for Soybean Improvement, Nanjing Agricultural University, Nanjing, China.

### Resistance Evaluation

The seedlings of 165 soybean accessions for association analysis were planted in round plastic pots (diameter × depth: 31 cm × 24 cm) filled with sand in an aphid-free greenhouse. About 45 seeds per each accession were planted in a pot. The planting dates were in May 2015, September 2015, May 2016, and September 2016 at the Jiangpu Experimental Station of Nanjing Agricultural University (32°12′N and 118°37′48″E) and these four experiments were denominated as environment 1, environment 2, environment 3, and environment 4. All accessions were planted with three biological replications and a randomized complete-block design was used for all field trials. After germination, the weak seedlings were pulled out. About 40 soybean plants per each genotype were inoculated with SMV strains of SC7 in every replication.

By using mortars and pestles, the fresh leaves of Nannong 1138-2 infected with SC7 were independently grinded in 0.01 mol/L sodium phosphate buffer (pH 7.2–7.4). Seedlings were manually inoculated with the inoculum by rubbing primary leaves at the V1 stage and inoculated again a week later. Disease symptoms were observed from day 7 to 1 month after inoculation. Resistant accessions were symptomless reaction to SMV infection and susceptible accessions were showed systematic mosaic leaves. The rate of susceptible plants to the total inoculated plants of each accession was defined as the disease rate (DR). The standard was according to Pu et al. (1983), DR of resistant accession was less than 10% and susceptible accession was greater than 10%.

### Phenotypic Data Analysis

Statistical analysis of the phenotypic data including descriptive statistics, analysis of variance (ANOVA), and broad heritability were using R software (R Development Core Team, 2015). The best linear unbiased prediction (BLUP) model in R (lme4 package) was used to estimate variation of year, line, and location components. The formula of linear unbiased prediction model was according to Merk et al. (2012)

$$Y_{\text{ik}}=\;\mu +G_{\text{i}}+Y_{\text{k}}+GY_{\text{ik}}+\;\varepsilon_{\text{ik}}$$

*Y*_ik_ indicates trait investigated, μ is total mean, *G*_i_ is genotypic effect of ith, *Y*_k_ is effect of kth year, *GY*_ik_ suggested genotype × year interaction, 𝜀_ik_ is residual error. The value of BLUP was produced from BLUP model with random effect and the BLUPs consisted of a new phenotype data was used for GWAS.

Broad-sense heritability (*h*^2^) of DR was calculated by using the method according to Kim et al. (2014) with the formula as below:

$$h^{2}=\sigma_{\text{g}}^{2}/(\sigma_{\text{g}}^{2}+\sigma_{\text{gy}}^{2}/n+\sigma_{\text{e}}^{2}/nr),$$

in the formula, 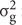 is genotype variance, 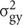 is interaction variance of genotype and year, 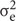 is variance of error components; *n* represent years; and *r* represent replications.

### SNP Genotyping and Genome-Wide Association Analysis

One hundred and sixty-five soybean mutants were genotyped by the 355 K SoySNP array (Wang J. et al., 2016). There were totally 282,469 SNPs were identified and the minor allele frequencies (MAFs) of these SNPs were evaluated by PLINK 1.07. After SNPs with MAFs < 0.05 were filtered out, 61,543 were left and used in genome-wide association analysis.

Difference models, including General Linear Model (GLM), GLM with Q (population structure), GLM with K (kinship), Mix Linear Model (MLM) with Q + K were selected and compared for the best fit model. The Bonferroni threshold as *P* ≤ (1/61543) or –Log_10_*P* ≥ 4.79 was used to define significant marker-trait associations.

### Population Structure Analysis

STRUCTURE 2.3.4 based on Bayesian model was adopted to analysis the population structure and relatedness in soybean. The length of burn-in period was set as 10,000 and number of Markov chain Monte Carlo (MCMC) replications after burn-in were set as 100,000. Hypothetic number of subpopulations (*K*) were set from 1 to 10 with three independent replications of operation. To evaluate the suitable *K* in this population, log likelihood of the LnP(D) and an *ad hoc* statistic Δ*k* based on the second-order rate of change of LnP(D) were analyzed for successive *k* (Evanno et al., 2005).

The 61,543 SNPs with MAFs < 0.05 were used to construct a neighbor-joining (NJ) phylogenetic tree with 1,000 bootstrap steps in Tassel 5.2.3. These SNPs were also used in PCA (principal component analysis) by PLINK 1.07 and population stratification was marked by different colors and shapes in a multidimensional scaling plot.

### Putative Genes Annotation and the Expression Analysis

In the GWAS results, we searched all the genes in the linkage disequilibrium (LD) decay distance of significantly associated SNPs and soybean genome annotation was referred to Glyma.Wm82.a1.v1.1^1^. Based on the soybean genomic annotations and bioinformatics, we predicted some putative genes related to resistance to SC7 within the LD decay distance of associated SNPs. Meanwhile, the amino acid sequences of the candidate genes in soybean response to pathogens stress were used to perform a BLASTP analysis in *Arabidopsis*.

To elucidate the expression of putative resistance genes, quantitative real-time PCR (qRT-PCR) was used to analysis their expression in the resistance and susceptible accessions, respectively. According to the average DR of four environments, we selected the extreme resistance and susceptible accessions, namely NJAU_M001 and NJAU_M082, respectively. The soybean seedlings were grown in an aphid-free greenhouse, and three replicated biological samples of leaves were collected in liquid nitrogen at 0, 2, 3, and 4 days after SC7 inoculating or mock inoculating. Total RNA of processed leaves were isolated with kit (TaKaRa, Japan). First strand cDNA synthesis kit (TaKaRa, Japan) was used to synthesis cDNA. With soybean tubulin gene (GenBank Number: AY907703) as an internal control, qRT-PCR was performed using ABI 7500 system (Applied Biosystems, United States) with following conditions: denaturing at 95°C for 5 min; 40 cycles at 95°C for 15 s, 60°C for 60 s. The fold changes of expression levels for each gene was calculated as ΔΔCT value. All the experiments were performed in three biological and three technical replicates.

## Results

### Phenotypic Variation of Disease Rate in Soybean

**FIGURE 1:**
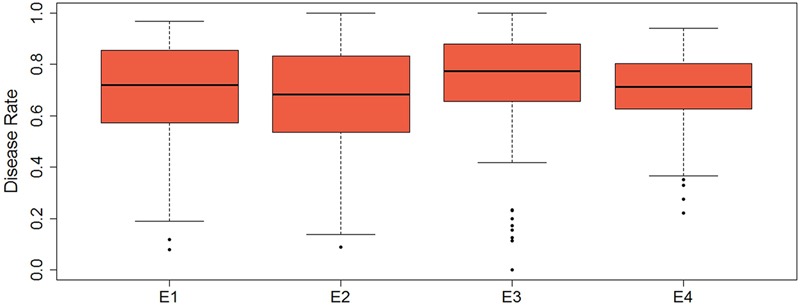
Box plot of the distribution of disease rate based on four environments data. The bold line indicates the median value, the red shaded area represents the lower and upper quartiles, and the dotted line range from edge line to upper edge line. Black dots were abnormal value.

**FIGURE 2:**
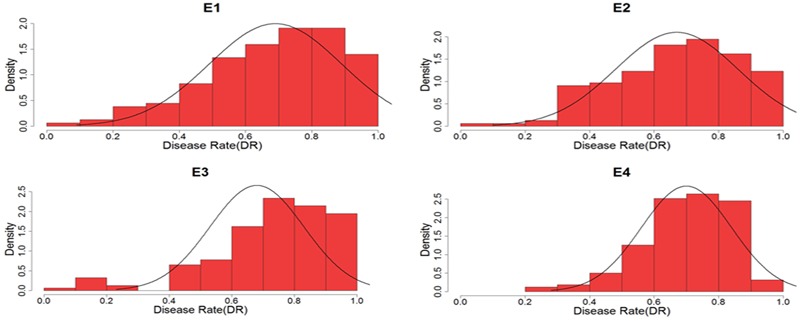
Density distribution of disease rate to soybean mosaic virus (SMV) strain of SC7 in four environments. E, environment.

Descriptive statistics, ANOVA, and broad heritability of DR in four environments were used to analyze the phenotypic data (**Table 1**). The average DRs in 165 soybean mutants were 0.68–0.70, which was visually showed on the box plots (**Figure 1**). As showed in **Figure 2**, DR of this population was stabled and without much difference among four environments. Coefficient of variation was ranged from 20.0 to 29.0%, indicating significant variation of DR among 165 soybean mutants. Moreover, the ANOVA suggested that the genotype effect and genotype × environmental interaction effects significantly influence DR (*P* ≤ 0.01). Broad-sense heritability for the DR was 60.6% in this population, which was a little lower than 86.4% previous reported by Yan et al. (2015). This result showed that the trait of DR can be stably inherited in soybean and some resistance accessions can be used in breeding.

**Table 1:** Descriptive statistics, analysis of variance (ANOVA) and broad heritability of disease rate in four environments.

Env	Mean	*SD*	Median	Min	Max	CV	Skew	Kur	G	G × E	*h*^2^
E1	0.69	0.2	0.72	0.08	0.97	29.0%	-0.77	0.04	^∗∗^	^∗∗^	60.6%
E2	0.67	0.19	0.68	0.09	1	28.3%	-0.49	-0.39	^∗∗^	^∗∗^	
E3	0.68	0.15	0.69	0.25	0.93	22.1%	-1.29	1.79	^∗∗^	^∗∗^	
E4	0.70	0.14	0.71	0.22	0.94	20.0%	-0.79	0.58	^∗∗^	^∗∗^	

*Env, environment; SD, standard deviation; Min, minimum; Max, maximum; CV, coefficient of variation; Skew, skewness; Kur, kurtosis; G, genotype; G × E, genotype × environment; *h*^*2*^, broad heritability; ^∗∗^, significant at *P* ≤ 0.01.*

### Population Structure Analysis, Linkage Disequilibrium, and Minor Allele Frequencies of Soybean Mutants

**FIGURE 3:**
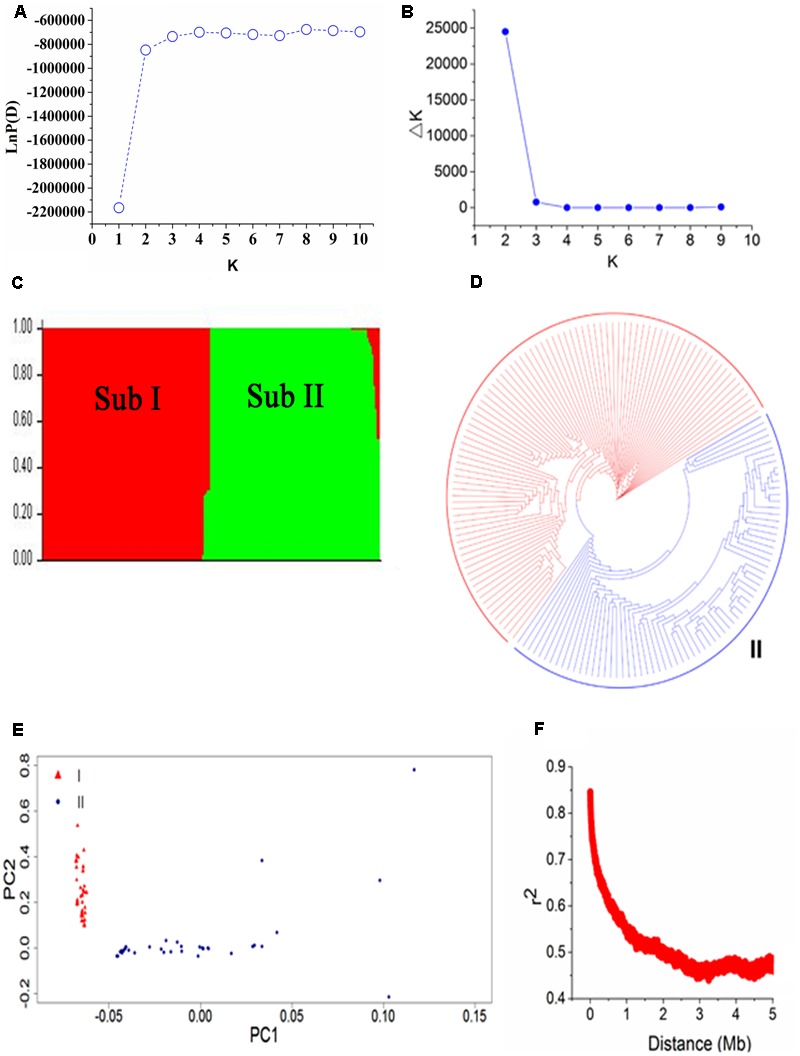
Population structure and linkage disequilibrium (LD) decay of 165 soybean accessions. **(A,B)** LnP(D) and Δ*k* based three repeats of STRUCTURE analysis. **(C)** The population structure estimated by STRUCTURE, there are two colored segments and each colored segment represents the percentage of the individual in the population. Sub means subpopulation. **(D)** A neighbor-joining tree of the 165 accessions that can be divided into two subpopulations. **(E)** Principal component analysis (PCA) of 165 soybean accessions, triangle in red represent Subpopulation I and dot with solid in blue is Subpopulation II. **(F)** LD decay based pair-wise *r*^2^ values in 5000-kb.

To evaluate the population structure and relatedness among 165 soybean mutants, we performed STRUCTURE, PCA, and constructed a NJ tree (**Figure 3**). In STRUCTURE results, LnP(D) was minimum at *K* = 1 and increased close to the maximum with the increased of *K* (**Figure 3A**). The *ad hoc* quantity (Δ*k*) showed a much higher possibility at *K* = 2 than at *K* = 3–10 among three replications in the program, suggested that the population can be clustered into two major subpopulations (**Figures 3B,C**): Subpopulation I and Subpopulation II. This result was further supported by the NJ tree and PCA (**Figures 3D,E**). Subpopulation I contains 93 accessions including the parent Nannong 86-4. And Subpopulation II contains 72 accessions including the parent Nannong 94-16. These results confirmed that accessions from the same ancient species have a close relationship with each other. Additionally, the Q-matrix at *K* = 2 calculated by STRUCTURE was used for subsequent genome-wide association analysis.

Linkage disequilibrium decay distance is an index for the suitable number of markers used in GWAS. A higher LD value indicates the need for a smaller number of markers and a lower LD value means the need for more markers. The LD decay plot based on pair-wise *r*^2^ values in 5000-kb was showed in **Figure 3F**. We found an obvious LD decay in this population. The average *r*^2^ values in the population were 0.506 in this study. The physical distance corresponding to half maximum of *r*^2^ values was 1 Mb.

**FIGURE 4:**
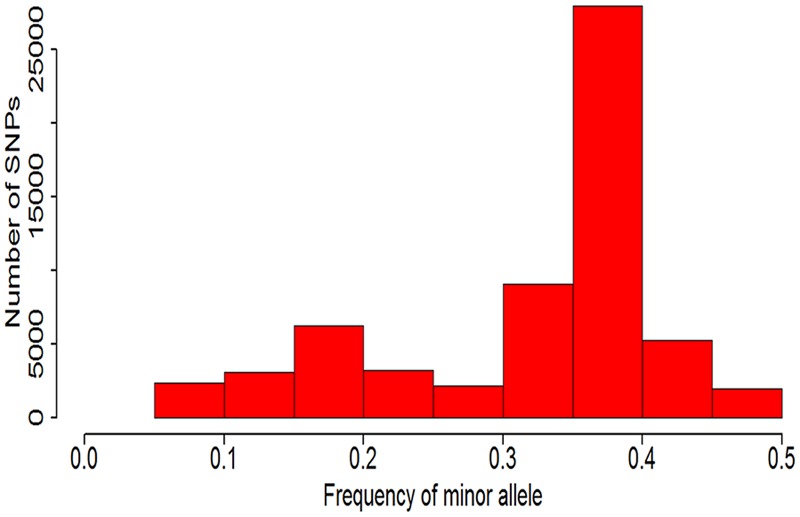
Minor allelic frequency distribution of the 165 soybean accessions based on 61,543 SNPs.

Because this soybean mutant population was mutagenic from two parent line (Nannong 86-4 and Nannong 94-16) and the probability of many accessions mutate in same point were very low. Approximately four fifths SNPs with MAFs less than 0.05 have been filtered out, and only 61,543 SNPs were left and used in this study. The distribution of the minor allelic frequencies of 61,543 SNPs were observed in **Figure 4** with continued distribution from 0.05 to 0.50. The average of MAF value of the 61,543 SNPs was 0.32 and 69.10% of the SNPs (42,528/61,543) had a MAF value between 0.30 to 0.45.

### Genome-Wide Association Analysis Reveals SNPs Significantly Associated with DR

**FIGURE 5:**
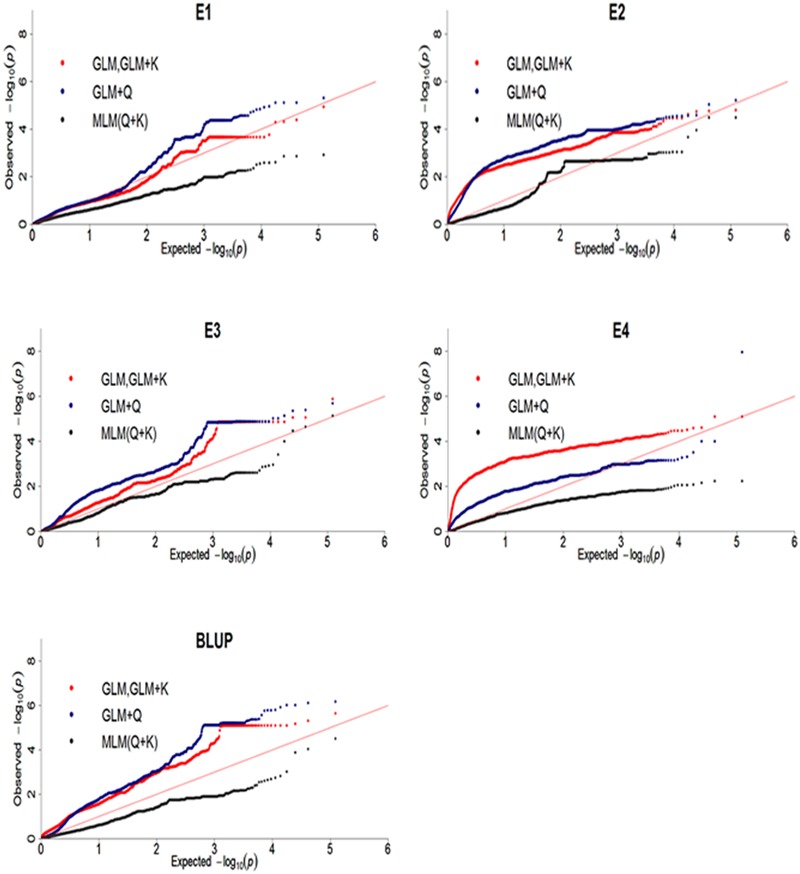
Quantile-quantile plots of three genome-wide association study (GWAS) models for disease rate in four environments and BLUP. E, environment; BLUP, best linear unbiased prediction.

To reveal SNPs significantly associated with DR, the genome-wide association analysis were performed. Difference models, including GLM, GLM with Q, GLM with K, MLM with Q + K were selected and compared for the best fit model. Results showed that MLM with Q + K excessive corrected observed *P*-value which lead to observed *P*-value less than expected *P*-value and no significantly associated SNP was identified (**Figure 5**). QQ plot of GWAS in GLM was same as GLM + K model which showed K matrix did not affect the result. Moreover, QQ plot of GLM model was much closer to diagonal line, suggesting that this model was more suitable than GLM + Q and MLM with Q + K models. Therefore, GLM model was selected in Tassel 5.2.3 (Bradbury et al., 2007) software in this study.

**FIGURE 6:**
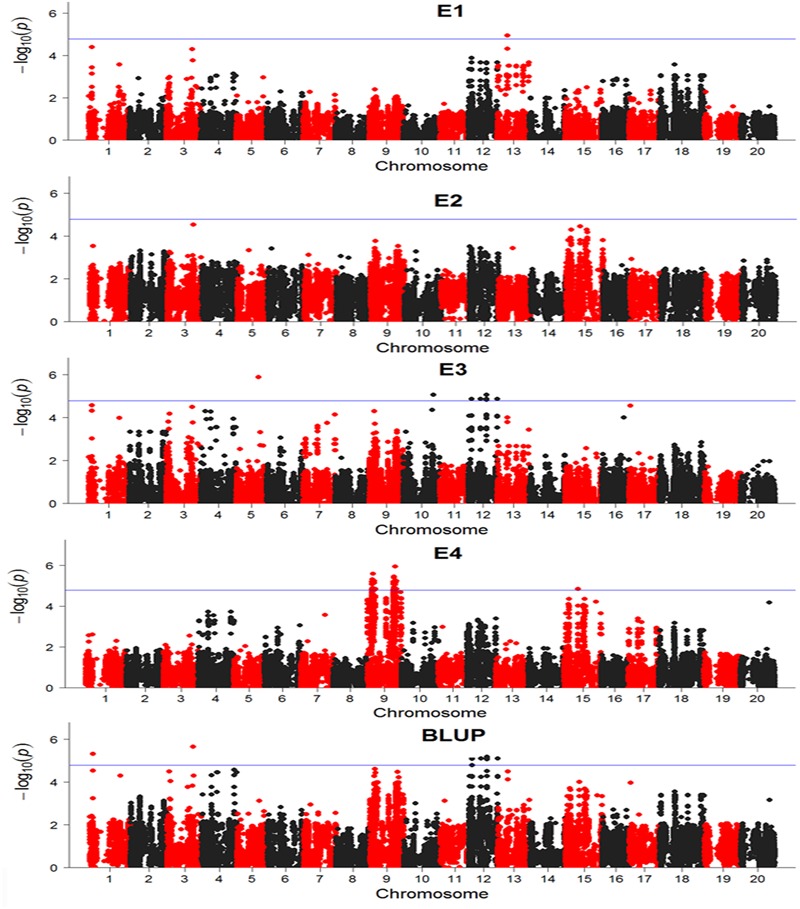
Manhattan plots base on General Linear Model (GLM) model in GWAS results. The horizontal line indicates a significant association signals (-log_10_*P* ≥ 4.79). E, environment; BLUP, best linear unbiased prediction.

There were totally 104 SNPs significantly associated with DR identified in four environments and BLUP (**Table 2**). Among these significant SNPs, 52 SNPs were repetitively detected in both E3 and BLUP on chromosome 12 (**Figure 6**). The rest SNPs were only detected in a single environment, which may be due to the influences of environments. On chromosome 9, two regions were found to be significantly associated with DR. Among these two regions, one region extending 1.6 Mb which contains 19 significant SNPs was overlapped with one QTL *SCN 39-5* (soybean cyst nematode) (Wu et al., 2009). The other region of chromosome 9, approximately 3.7 Mb long with 27 significant SNPs contains one QTL *Sclero 8-3* that resists to *Sclerotinia Stem Rot* (Vuong et al., 2008). Similar to chromosome 9, on chromosome 12, there were also two regions identified with strong signals associated with DR, which include 20 SNPs and 30 SNPs distributed in 111 and 170 kb physical area, respectively. Several QTLs reported to response to biotic stress, such as *Phytoph 9-1* (Wang et al., 2010), *Corn earworm 2-2* (Rector et al., 1999), *Pod borer 1-1* (Zhao et al., 2008), and *SCN 10-5* (Qiu et al., 1999) were mapped to this region. Additionally, Several SNPs distributed on other chromosomes, such as chromosome 1, 3, 5, 10, 13, and 15. In the vicinity of these identified SNPs, there were some QTLs reported to response to biotic stress. For example, *SCN 19-1, SCN 19-2, SCN 19-4, SCN 33-7, SCN 44-5, SCN 18-1*, and *SCN 37-1* have been reported to resistant to soybean cyst nematode (Yue et al., 2001; Guo et al., 2006; Vuong et al., 2010; Jiao et al., 2015); SDS 13-1 was associated with resistant to soybean fungi disease sudden death syndrome (SDS) (Abdelmajid et al., 2012); *Japanese beetle resistance 1-4* was associated with herbivory pest Japanese beetles resistance (Yesudas et al., 2010); *Phytoph 5-3* was related to *Phytophthora sojae* infection (Wu et al., 2011); *Rag 3-2* associated with aphid resistance(Zhang et al., 2009); *Asian Soybean Rust 2-4* resisted to fungal pathogen *Phakopsora pachyrhizi* (Harris et al., 2015); *Peanut root-knot nematode 2-2* was associated with resistance to peanut root-knot nematode in soybean (Tamulonis et al., 1997).

**Table 2:** SNPs loci associated with disease rate (DR) identified via a genome-wide association study (GWAS) in 165 soybean accessions.

Chr.^a^	MSS^b^ position	MSS *P*-value	No. of SNP^c^	Significant region^d^		QTLs	References	Env.^e^
				Start	End			
1	4660270	4.8305E-06	1	3666713	5653827	*SCN 19-1*^f^*; SCN 19-2*^f^*SCN 19-4*^f^*; SDS 13-1*^g^	Yue et al., 2001; Abdelmajid et al., 2012	BLUP
3	35857652	2.1768E-06	1	34864095	36851209	*SCN 33-7*^f^*; SCN 44-5*^f^	Guo et al., 2006; Jiao et al., 2015	BLUP
5	30283423	1.32E-06	1	29289866	31276980	*SCN 18-1*^f^*; Japanese beetle resistance 1-4*	Yue et al., 2001; Yesudas et al., 2010	E3
9	6502417	8.22E-06	19	5540581	9474855	*SCN 39-5*^f^	Wu et al., 2009	E4
9	37738517	1.3E-05	27	35253874	39962448	*Sclero 8-3*^h^	Vuong et al., 2008	E4
10	41634779	8.43E-06	1	40641222	42628336	*SCN 37-1*^f^*; Phytoph 5-3*^i^	Vuong et al., 2010; Wu et al., 2011	E3
12	4444659	7.8E-06	20	3906872	5018746	*Phytoph 9-1*^i^	Wang et al., 2010	E3, BLUP
12	16574555	1.35E-05	1	15580998	17568112	*Corn earworm 2-2*	Rector et al., 1999	E3, BLUP
12	25126021	7.8E-06	30	24569981	25740186	*Pod borer 1-1*	Zhao et al., 2008	E3, BLUP
12	40030784	7.6E-06	1	39037227	41024341	*SCN 10-5*^f^	Qiu et al., 1999	E3, BLUP
13	14139325	1.11E-05	1	13145768	15132882	*Rag 3-2*^j^	Zhang et al., 2009	E1
						*Asian Soybean Rust 2-4*		
15	18849136	1.41E-05	1	17855579	19842693	*Peanut root-knot nematode 2-2*	Tamulonis et al., 1997; Harris et al., 2015	E4

*^a^ Chromosome; ^b^ most significant SNP; ^c^ number of significant SNPs; ^d^ significant region was in the LD decay distance flanking the significant SNPs; ^e^ environment; ^f^ soybean cyst nematode; ^g^ sudden death syndrome; ^h^ Sclerotinia Stem Rot; ^i^*Phytophthora sojae*; ^j^ aphid resistance.*

These results showed that SNPs significantly associated with DR were also mapped to the disease and pest resistance loci reported before. It suggested that these loci might be common in the resistance to biotic stress and that these loci were valuable for marker-assisting selection in breeding.

### Identification of Putative Genes and the Expression Analysis

To annotate the SNPs significantly associated with resistance to SC7, we searched the 1 Mb (LD decay distance) flanked regions of these SNPs to find putative genes. Then we utilized the soybean genome annotation information and performed BLASTP against *Arabidopsis* genome to annotate the putative genes (**Table 3**). On chromosome 1, *Glyma.01G043300* was a putative gene which was homologous to *WRKY72* in *Arabidopsis*. The latter was reported to contribute to the basal immunity and defense against root-knot nematodes (Bhattarai et al., 2010). Moreover, *AtWRKY72* can also utilize SA-independent defense mechanisms (Bhattarai et al., 2010). On chromosome 9, *Glyma.09g063800* was an eukaryotic translation elongation factor EF1B and contained guanine nucleotide exchange domain (Andersen et al., 2000) and a GST C-terminal domain (Dixon et al., 2002). *Glyma.09g063800* was homologous to *eEF1Bβ* in *Arabidopsis*. *eEF1Bβ* was reported to facilitate potato virus X infection and interacted with PVX *TGBp1* (Hwang et al., 2015). In soybean, Luan et al. (2016) reported that knocked down the four *GmEF1B* homologs *Glyma.13g07320*0, *Glyma.02g276600, Glyma.14g039100*, and *Glyma.04g195100* enhanced resistance against the virulent G7 strain of SMV. On chromosome 12, we identified a receptor-like protein *Glyma.12g233700* which was homologous to *AtRLP9* in *Arabidopsis*. *AtRLP9* was reported to co-regulate with Rust-Induced Secreted Protein (RISP), which was strong and specific induced by an avirulent strain of *Melampsora larici-populina* (Rinaldi et al., 2007). The gene expression analysis in response to downy mildew showed that *AtRLP9* was distributed in pathology-related group which also contained AtRLP52, AtWRKY72, and other NBS-LRR class disease resistance protein (Coker et al., 2015). *AtRLP52* was found to be involved in the resistance response (Ramonell et al., 2005). Therefore, these three genes (*Glyma.01G043300, Glyma.09g063800*, and *Glyma.12g233700*) were identified as putative genes related to SC7 resistance.

**Table 3:** Putative genes associated with resistance to SC7.

Chromosome	Reported QTL	Gene orthologs in *Arabidopsis*	Gene ID	Gene annotation
1	*SCN 19-1 SCN 19-2 SCN 19-4 SDS 13-1*	*WRKY72*	*Glyma.01G043300*	WRKY transcription factor; compartment: nucleus; defense response; regulation of transcription, DNA-dependent
9	*SCN 39-5*	*eEF1Bβ*	*Glyma.09g063800*	Translation elongation factor EF1B/ribosomal protein S6 family protein; EF-1 guanine nucleotide exchange domain
12	*SCN 10-5*	*AtRLP9*	*Glyma.12g233700*	Receptor-like protein 9; signal transduction; leucine rich repeat N-terminal domain; disease resistance protein.

**FIGURE 7:**
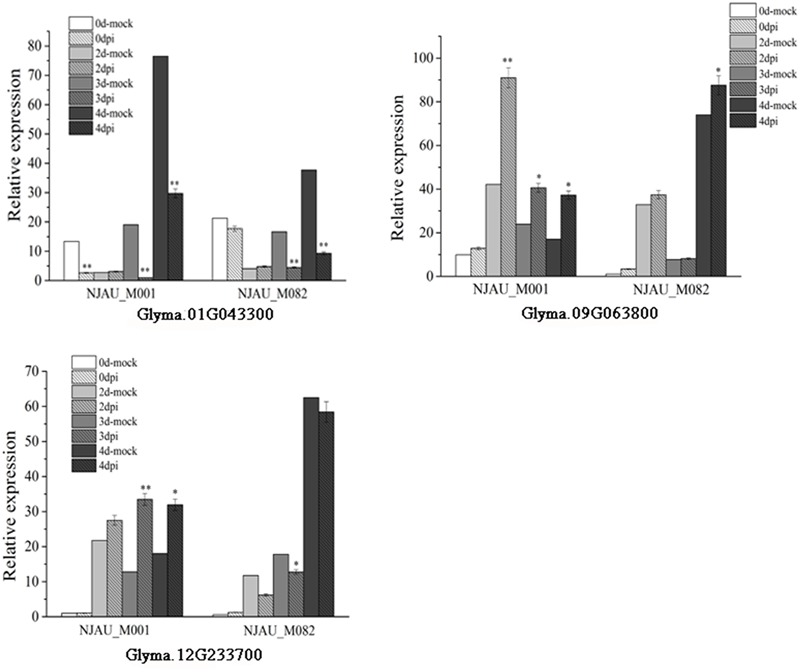
Expression analysis of the three putative genes in SMV resistant soybean NJAU_M001 and SMV susceptible soybean NJAU_M082. In the legend, 0, 2, 3, and 4 days-mock represent 0, 2, 3, and 4 days after inoculating with 0.01 mol/L sodium phosphate buffer, respectively. Dpi means days past inoculated with SC7. Error bar indicate the standard deviation. Results are representative of three biological replicates. ^∗^, significant at *P* ≤ 0.05; ^∗∗^, significant at *P* ≤ 0.01.

Expression of putative genes were analyzed through qRT-PCR at 0, 2, 3, and 4 days after SC7 or mock inoculated in a resistance (NJAU_M001) accession and a susceptible (NJAU_M082) accession. As shown in **Figure 7**, the expression of *Glyma.01G043300* significantly decreased with SC7 inoculating than mock inoculating, which was consistent in both the resistance and susceptible accessions. On the contrary, the *Glyma.09g063800* was significantly up-regulated in the SMV resistance NJAU_M001 soybean at 2, 3, and 4 days. And the expression level was the highest at 2 days after SC7 inoculating compared with mock inoculating in the same time. Meanwhile, in SMV susceptible soybean NJAU_M082, the expression of *Glyma.09g063800* increased slightly at 0, 2, and 3 days and significantly increased at 4 days after SC7 inoculating compared with mock inoculating. The expression pattern of *Glyma.12g233700* was different in the SMV resistant and susceptible soybean accessions. The expression levels of *Glyma.12g233700* up-regulated in the SMV resistant soybean NJAU_M001, and decreased in SMV susceptible soybean NJAU_M082 after SMV inoculating compared with mock inoculating. These expression analyses indicate the three putative genes were induced by the infection of SC7, which should provide an important basis for further functional study of these SMV putative resistance genes.

## Discussion

### Resistance in Development to SMV Was a Quantitative Trait That Ubiquitous in Soybean

Soybean mosaic virus is one of the serious diseases that cause severe yield loss in soybean. The spread of SMV disease is by aphid. Although chemical pesticide can kill aphid and interdict the diffuse of SMV, it can also increase the cost to the farmer and cause environment pollution. Many studies have been reported on inheritance of resistance in infection to SMV and three distinct dominant SMV resistant loci, namely *Rsv1, Rsv2*, and *Rsv4* have been mapped in PI 96983, OX686, and V94-5152, respectively (Kiihl and Hartwig, 1979; Buzzell and Tu, 1989; Hayes et al., 2000). In addition to resistance in infection to SMV, Zhi et al. (2005) reported another genetic mechanism in resistance to SMV, which was called resistance to SMV extension or development. And the study showed that the resistance in development to Sa strains of SMV was controlled by one additive major gene plus additive-dominant polygenes (Zhi et al., 2005). Guo et al. (2012) also reported resistance genetic mechanism in development to SC3 was controlled by two equality-additive major genes. Up to now, many researchers have focused on the resistance to SMV infection, however, few studies have been focused on the resistance to SMV extension. Although the phenotypic reaction in resistance to SMV extension was not obvious than that to SMV infection, it was more extensive and durable in resistance to SMV. Accordingly, it is important to identify the loci that control the resistance to SMV extension. In this study, we treated SMV resistance as a quantitative trait and used DR which showed a roughly normal distribution (**Figure 2**, skewness and kurtosis as shown in **Table 1**) as the phenotypic data. Then we used GWAS to find putative resistance loci. These loci would provide a basis for further functional study and be applied in the breeding of resistant cultivars.

### Linkage Disequilibrium in Soybean Mutant Population

Genome-wide association study was based on LD, which was different in various crops and populations, moreover, LD could be broken by historical recombinants (David et al., 2001). In maize, the LD decay was estimated to be ≤2 kb in diverse inbred lines, because maize was a kind of cross-pollination species (Michael et al., 2009; Yang et al., 2010). In rice, the LD decay distance of *indica* and *japonica* were ranged from 100 to 200 kb (Mather et al., 2007). In soybean, as a kind of self-pollination species, the LD decay distance was evaluated approximately 500 kb which was longer than other cross-pollination crops (Hyten et al., 2007). Moreover, LD was different in wild and cultivated soybeans, Wang J. et al. (2016) reported that the distance over which LD decays to half of its maximum value was 80 kb in wild soybeans and 130 kb in cultivated soybeans. In this study, the average extension of LD decay distance for the population consisting of 165 soybean mutants was estimated about 1000 kb, which was larger than the previously reported value in the natural population (Wang J. et al., 2016). The reasons for the high value LD in this study may result from the close genetic relatedness and a relatively small population size. By contrast, the natural population consists of different landraces from different geographic origins with phenotypic variations. Different geographic landraces and far genetic relatedness with each other result in a lower LD level comparing with near-isogenic accessions. The high LD level in soybean indicates the need for a small amount of markers in GWAS to identify SNPs associated with target trait, which would facilitate marker-assisted selection in breeding.

### Novel Loci Identified in Resistance to SMV

Based on GLM model, we identified 104 SNPs significantly associated with resistance to SMV in four environments and BLUP. Some of these SNPs were located in or near previous reported QTLs that have been mapped by linkage analysis for resistance to SMV, soybean cyst nematode, *Sclerotinia Stem Rot*, fungal pathogen *Phakopsora pachyrhizi*, and *Phytophthora sojae* (Yue et al., 2001; Guo et al., 2006; Vuong et al., 2008, 2010; Wu et al., 2011; Harris et al., 2015; Jiao et al., 2015; Yan et al., 2015), suggesting these SNPs could be related with biotic stress. Nevertheless, half of these significant SNPs were only detected in a single environment. For example, on chromosome 1 and 3, some SNPs associated with resistance to SMV were detected in E1, E3, and BLUP, but only in BLUP the SNPs reached the significant level. Similarly, on chromosome 15, some SNPs in E2, E4, and BLUP were detected but few of them reached the significant level. These results may be result from a high threshold as *P* ≤ (1/61543) or -Log_10_*P* ≥ 4.79 set in this study. If we decreased the threshold, these SNPs would be repetitively identified in two environments at least. These results indicated that some resistance genes might exist in these genomic regions and the SNP markers we identified would helpful for assembling these genes to improve resistance to SMV.

## Conclusion

In this study, the high-density SNP markers and a soybean mutant panel were used in GWAS to discover new resistance loci to SMV. In GWAS results, 104 SNPs significantly associated with DR to SMV. Many novel SNPs associated with the resistance to SMV were identified on chromosome 1, 3, 5, 9, 10, 12, 13, and 15. These novel SNPs located in or near biotic stress QTLs that previously reported. Then the genetic annotation and expression analyses identified three putative genes were relevant with resistance to SMV and were worthy of further investigation. Our results will provide insights for improving marker-assisted selection and molecular designed breeding strategies for new accession with resistance to SMV.

## Author Contributions

This study was designed by HC and DY. SNP markers were analyzed by JW. ZC conducted the experiments, including phenotypic data evaluation and GWAS analysis. ZC wrote this manuscript. JW, DY, and HL revised the manuscript. FY, YY, LW, PZ, and JD provided input in experiments. All authors read and approved the final version to be published.

## Conflict of Interest Statement

The authors declare that the research was conducted in the absence of any commercial or financial relationships that could be construed as a potential conflict of interest. The reviewer PS and handling Editor declared their shared affiliation.

## Footnotes

1 www.phytozome.net
